# Supplementary figures and images for: A new paradigm for leprosy diagnosis based on host gene expression
Source: PLoS Pathog. 2021 Oct 25;17(10):e1009972. doi: 10.1371/journal.ppat.1009972 (PMC8568100; doi:10.1371/journal.ppat.1009972)

# A

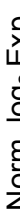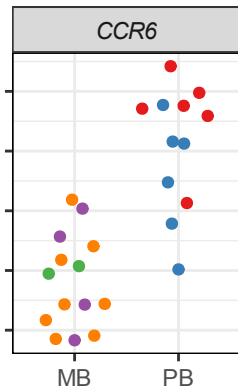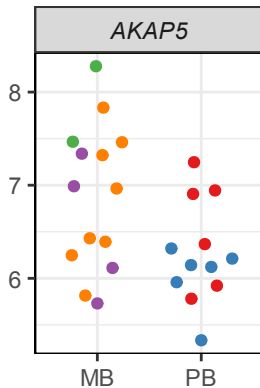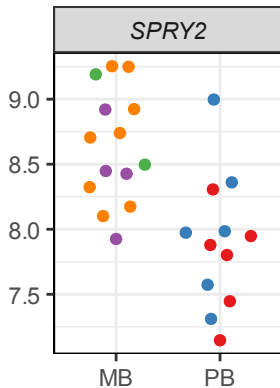

**B**

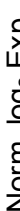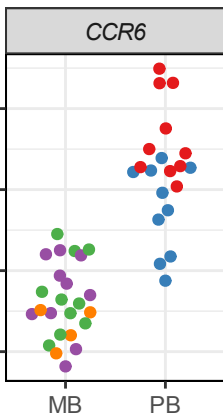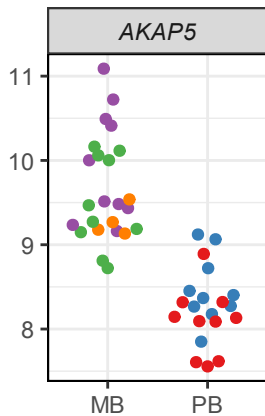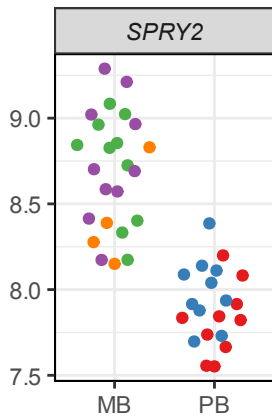

Group    ● TT    ● BT    ● BB    ● BL    ● LL

Supplement: S1 Fig — Normalized log2 expression values per group from (A) this study RNA-seq dataset or (B) Belone et al. (GSE74481) [24]. The genes shown were selected in 25%–50% of the LASSO models (Fig 4B) according to the bootstrap. MB, multibacillary leprosy; PB, paucibacillary leprosy; TT, tuberculoid leprosy; BT, borderline-tuberculoid; BB, borderline-borderline; BL, borderline-lepromatous; LL, lepromatous. Each point represents an independent skin biopsy from a patient. Y-axis values are not comparable between panels A and B. (PDF) [file ppat.1009972.s002.pdf]

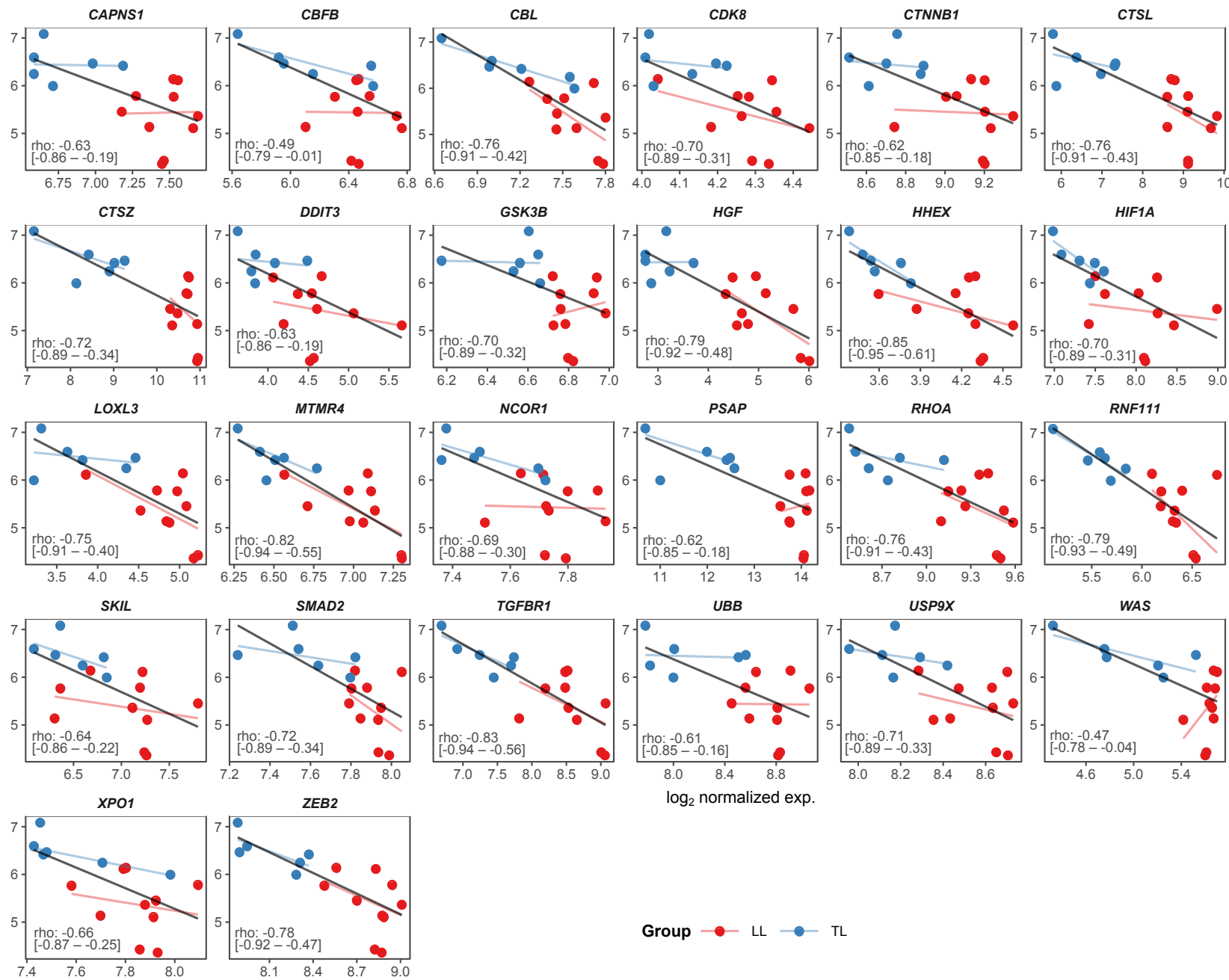

Supplement: S2 Fig — Scatter plots of scores (average normalized log2 expression) calculated from genes with previously documented down-regulation in leprosy skin lesions against dedifferentiation-related genes with Montoya et al. RNA-seq dataset (GSE125943) [28]. Lines were drawn based on intercept and beta estimates from robust linear regression for all samples (black) or separately for TL (tuberculoid leprosy, blue), and LL (lepromatous leprosy, red). X-axis shows log2 normalized expression values. Spearman’s rho are shown along with nominal 95% confidence intervals inside the plots. Most genes shown have FDR < 0.1 and rho ≤ -0.6. Related to Fig 6. (PDF) [file ppat.1009972.s003.pdf]

Spearman's rho

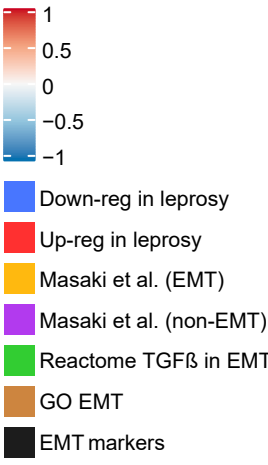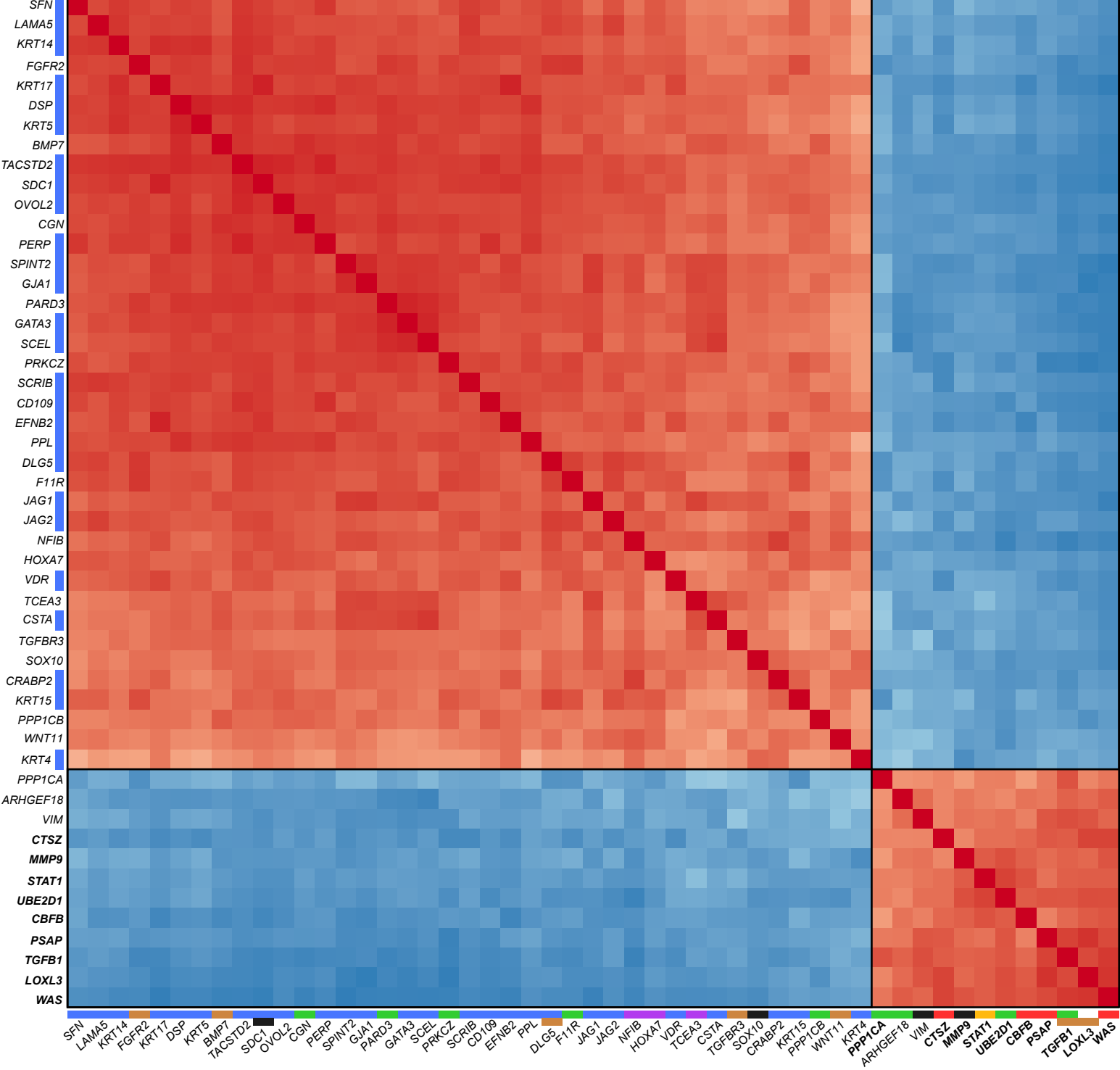

Supplement: S3 Fig — Heat plot with Spearman’s rho correlation coefficient of the strongest correlations from all ontologies screened after multiple testing adjustment (BH-FDR). Most genes shown have FDR ≤ 0.0001 and rho ≤ -0.7. Bottom colored rectangles indicate which category the gene was present (some genes co-occur). Related to Fig 6. (PDF) [file ppat.1009972.s004.pdf]
